# The application of diode laser in the treatment of oral soft tissues lesions. A literature review

**DOI:** 10.4317/jced.53795

**Published:** 2017-07-01

**Authors:** Daniel Ortega-Concepción, Jorge A. Cano-Durán, Juan-Francisco Peña-Cardelles, Víctor-Manuel Paredes-Rodríguez, José González-Serrano, Juan López-Quiles

**Affiliations:** 1 Department of Oral Medicine and Surgery, School of Dentistry, Complutense University, Madrid, Spain

## Abstract

**Background:**

Since its appearance in the dental area, the laser has become a treatment of choice in the removal of lesions in the oral soft tissues, due to the numerous advantages they offer, being one of the most used currently the diode laser. The aim of this review was to determine the efficacy and predictability of diode laser as a treatment of soft tissue injuries compared to other surgical methods.

**Material and Methods:**

A literature review of articles published in PubMed/MEDLINE, Scopus and the Cochrane Library databases between 2007 and 2017 was performed. “Diode laser”, “soft tissue”, “oral cavity” and “oral surgery” were employed for the search strategy. Only articles published English or Spanish were selected.

**Results:**

The diode laser is a minimally invasive technology that offers great advantages, superior to those of the conventional scalpel, such as reduction of bleeding, inflammation and the lower probability of scars. Its effectiveness is comparable to that of other types of lasers, in addition to being an option of lower cost and greater ease of use. Its application in the soft tissues has been evaluated, being a safe and effective method for the excision of lesions like fibromas, epulis fissuratum and the accomplishment of frenectomies.

**Conclusions:**

The diode laser can be used with very good results for the removal of lesions in soft tissues, being used in small exophytic lesions due to their easy application, adequate coagulation, no need to suture and the slightest inflammation and pain.

** Key words:**Diode laser, soft tissues, oral cavity, oral surgery.

## Introduction

Benign hyperplasias and tumors of the oral soft tissues are lesions that occur with an increase in volume, being traumatic and inflammatory the most frequent causes. They consist of certain habits, dental or mucous infections or prosthetic traumatisms that become aggressive agents for the epithelial and connective tissues, which react producing hyperplasias. The clinical history plays a fundamental role to establish the diagnosis. If there is an etiology of constant trauma or infection, the diagnosis will be inclined to a reactive hyperplasia, whereas if these causes are not present, a true tumor must be suspected (1,2).

When these lesions require a surgical treatment, different procedures as conventional scalpel, electric scalpel or different types of lasers can be used. Advances in laser technology, as well as a better understanding of the different systems, have allowed to extend the clinical use of this instrument in dentistry, being used for minimally invasive conservative dental treatments, autoimmune diseases or surgical treatments (3).

The most commonly used lasers in oral surgery are high power lasers as CO2 laser, the Erbium family lasers, the Nd:YAG laser and the diode laser, which are mainly used for the elimination of the tissues (4,5).

The CO2 laser was the first laser to appear in Dentistry, thanks to its excellent cutting ability, being still a very employed and effective method. Its appearance was a very important advance in the development of surgical lasers. Currently it is applied due to its advantages for a correct tissue incision, coagulation or postoperative benefits (6).

Diode laser has become very popular in dentistry due to its small size and ease of use for minor soft tissue surgery (6). Based on its photothermal effect, it is used for the removal of small lesions of the oral mucosa by excision or vaporization procedures (7,8).

The aim of this literature review is to evaluate, based on available scientific literature, the efficacy and predictability of diode laser in the treatment of soft tissue lesions, comparing it with other surgical methods such as conventional scalpel or other types of laser.

## Material and Methods

-Search Strategy.

A comprehensive search of the literature was conducted until January 31st, 2017 in the following databases: Pubmed/MEDLINE, Scopus and the Cochrane Library. The search strategy used the following combination of terms: Diode laser AND soft tissues OR oral cavity OR oral surgery. An additional hand search to find potential eligible studies was performed.

-Study Selection

Inclusion Criteria. Full-text articles published between 2007 and 2017 were included. Only full text articles published in English or Spanish language were selected. Clinical trials and case series that used high power diode laser evaluating histologic or clinical variables were chosen. At first, only humans studies were selected, however, due to the lack of comparative studies, animal studies were also included.

Exclusion Criteria. Articles published before 2007, in a language other than English or Spanish or not available in full text were not included. Studies evaluating other types of lasers or low power diode laser and studies with no control group were also excluded.

**Figure 1:**
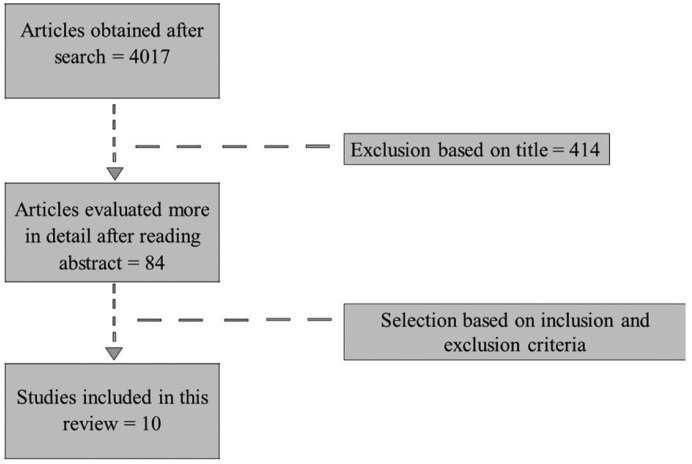
Flow diagram of the literature search.

The response to the search strategy yielded 4017 results. Then, 2 independent researchers reviewed all the titles and articles that did not use high power diode laser in the treatment of oral soft tissues lesions were discarded, obtaining 414 potentially selectable papers. After reading the abstract, 84 of them were selected for a more detailed evaluation. Finally, 10 full text studies were included, excluding the rest of them for not meeting the established inclusion criteria (Fig. 1).

## Results

Main Findings.

All the articles found the diode laser to be effective in the extirpation of lesions in the oral soft tissues. A total of four studies (9-12) compared the effectiveness of the diode laser with respect to the conventional scalpel, concluding in a clear superiority of the laser. Four articles (13-16) compared the CO2 laser with the diode laser in the treatment of exophytic lesions in oral soft tissues. In relation to the Erbium family lasers, four articles (12,14,15,17) discussed their use in oral surgery for the elimination of exophytic lesions. One study (15) compared the efficacy of Nd:Yag laser compared to other lasers in the removal of oral lesions and also, only one study compared the diode laser with the electric scalpel (18). The indications found for the use of these lasers are summarized in  Table 1.

**Table 1:**
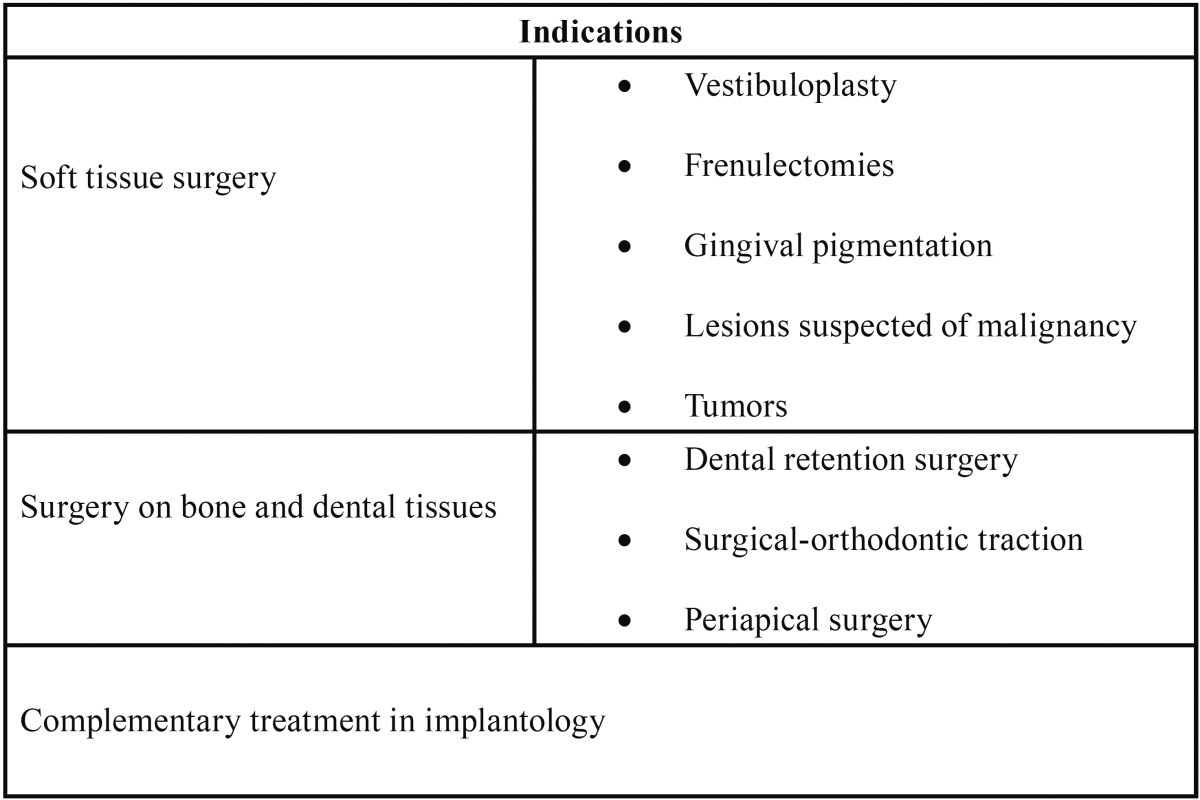
Indications for high power lasers.

## Discussion

One of the applications of lasers in dentistry is the extirpation of soft tissue lesions. Today, the diode laser is being one of the most employed, as it provides numerous advantages compared to the conventional scalpel or to other types of lasers.

The superiority of the diode laser in relation to the conventional scalpel offers no doubts. D’Arcangelo *et al.* (9) and Amaral *et al.* (10) compared both techniques concluding that diode laser offers numerous advantages compared to the conventional scalpel as a lower intraoperative bleeding, a lower swelling of the area, better coagulation and scarring, no need of suture, reduction of surgical time and lesser degree of postsurgical pain. In addition, the laser instantly disinfects the surgical wound, and lesser further mechanical trauma is also confirmed by Bakhtiari *et al.* (11) study. On the other hand, Jin *et al.* (12) reported that diode laser produced greater tissue damage compared to the conventional scalpel and the Er Cr: YSGG laser.

The CO2 laser was the first laser introduced in dentistry with excellent cutting precision, and thus, performing less invasive surgery (7). It offers the same advantages compared to the conventional scalpel as the diode laser. Moreover, Suter *et al.* (13) reported a lower collateral thermal damage at the edges of the lesion with this laser, findings that match with the study of Cercadillo-Ibarguren *et al.* (14) and Azevedo *et al.* (15). Monteiro *et al.* (16) considered it as the Gold Standard for epulis fissuratum extirpation due to its speed and greater penetration. However, its cost, clearly superior to the diode laser, and its greater difficulty of handling due to its big size, make it a method difficult to apply in the clinical practice.

Erbium lasers (Er: YAG and Er, Cr: YSGG) have been recently used in dentistry for the development of dental cavities and removal of bone tissue (5). Like the CO2 laser, its length wave allows it to penetrate less into the tissue, so the degree of injury on the treated area is lower when compared to the diode laser and therefore its recovery is faster (12,14,15,17). However, Fekrazad *et al.* (17) found that the degree of intraoperative coagulation and hemostasis that it grants with respect to the diode laser is lower and, in addition, its mechanism of pulsatile emission results in a more unequal cut than the continuous-wave lasers, which is very important in the removal of lesions in aesthetic areas. The price is also higher compared to the diode laser (17,19).

Regarding Nd:YAG lasers, these are frequently used for cartilage, bone, tattoo or hair remotion (5). Its wavelength and its ability to penetrate deep in the soft tissues, creates significant collateral damages in the tissues far superior to the rest of lasers (15). Asnaashari *et al.* (4) showed it desirable for vascular injuries, but not for other pathologies. Therefore, they are not used in the removal of exophytic soft tissue lesions, as the diode laser can be a safer alternative to them (15,20).

Among the mentioned applications of the laser, we also found the removal of gingival melanosis. A comparative study of the use of 980 nm diode laser and electric scalpel for the treatment of gingival hyperpigmentation revealed statistically significant results for the laser in postoperative pain during the first 24 hours (18). The patients in the laser group experienced less pain compared to the electric scalpel group, which can be attributed to the analgesic effects of these diode lasers, due to a disruption of the sodium and potassium pumps in the cell membrane, thus producing a loss of impulse conduction, although it is also considered the possibility that it is related to the ablation of the nerve endings. Although there are enough studies confirming a greater advantage of the laser compared to other techniques, we should consider that certain studies have shown recurrences in the treatment of lesions such as leukoplakia after removal with a laser (21).

The diode laser can be used with very good results in oral surgery for the removal of soft tissue lesions, being especially used in small exophytic lesions. Due to its easy application and low cost, adequate coagulation, no need to suture, less inflammation and pain, low time of treatment, better repair and recovery and the rare intraoperative and postoperative complications, is an effective and predictable method when performing surgeries in oral soft tissues, clearly superior to the conventional scalpel and with numerous advantages over other types of lasers. Therefore, diode lasers are becoming a tool present in routinary clinical practice. However, further comparative studies are still needed, mainly to assess their long-term efficacy.

## References

[1] Torres-Domingo S, Bagan JV, Jiménez Y, Poveda R, Murillo J, Díaz JM (2008). Benign tumors of the oral mucosa: a study of 300 patients. Med Oral Patol Oral Cir Bucal.

[2] Allon I, Kaplan I, Gal G, Chaushu G, Allon DM (2014). The clinical characteristics of benign oral mucosal tumors. Med Oral Patol Oral Cir Bucal.

[3] Asnaashari M, Safavi N (2013). Application of Low level Lasers in Dentistry (Endodontic). J Lasers Med Sci.

[4] Asnaashari M, Zadsirjan S (2014). Application of laser in oral surgery. J Lasers Med Sci.

[5] Deppe H, Horch HH (2007). Laser applications in oral surgery and implant dentistry. Lasers Med Sci.

[6] Azma E, Safavi N (2013). Diode laser application in soft tissue oral surgery. J Lasers Med Sci.

[7] Desiate A, Cantore S, Tullo D, Profeta G, Grassi FR, Ballini A (2009). 980 nm diode lasers in oral and facial practice: current state of the science and art. Int J Med Sci.

[8] Sotoode SM, Azimi S, Taheri SA, Asnaashari M, Khalighi H, Rahmani S (2015). Diode Laser in Minor Oral Surgery: A Case Series of Laser Removal of Different Benign Exophytic Lesions. J Lasers Med Sci.

[9] D'Arcangelo C, Di Nardo Di Maio F, Prosperi GD, Conte E, Baldi M, Caputi S (2007). A preliminary study of healing of diode laser versus scalpel incisions in rat oral tissue: a comparison of clinical, histological, and immunohistochemical results. Oral Surg Oral Med Oral Pathol Oral Radiol Endod.

[10] Amaral MB, de Ávila JM, Abreu MH, Mesquita RA (2015). Diode laser surgery versus scalpel surgery in the treatment of fibrous hyperplasia: a randomized clinical trial. Int J Oral Maxillofac Surg.

[11] Bakhtiari S, Taheri JB, Sehhatpour M, Asnaashari M, Attarbashi Moghadam S (2015). Removal of an Extra-large Irritation Fibroma With a Combination of Diode Laser and Scalpel. J Lasers in Med Sci.

[12] Jin JY, Lee SH, Yoon HJ (2010). A comparative study of wound healing following incision with a scalpel, diode laser or Er,Cr:YSGG laser in guinea pig oral mucosa: A histological and immunohistochemical analysis. Acta Odontol Scand.

[13] Suter VG, Altermatt HJ, Sendi P, Mettraux G, Bornstein MM (2010). CO2 and diode laser for excisional biopsies of oral mucosal lesions. A pilot study evaluating clinical and histopathological parameters. Schweiz Monatsschr Zahnmed.

[14] Cercadillo-Ibarguren I, España-Tost A, Arnabat-Domínguez J, Valmaseda-Castellón E, Berini-Aytés L, Gay-Escoda C (2010). Histologic evaluation of thermal damage produced on soft tissues by CO2, Er,Cr:YSGG and diode lasers. Med Oral Patol Oral Cir Bucal.

[15] Azevedo AS, Monteiro LS, Ferreira F, Delgado ML, Garcês F, Carreira S (2016). In vitro histological evaluation of the surgical margins made by different laser wavelengths in tongue tissues. J Clin Exp Dent.

[16] Monteiro LS, Mouzinho J, Azevedo A, Câmara MI, Martins MA, La Fuente JM (2012). Treatment of epulis fissuratum with carbon dioxide laser in a patient with antithrombotic medication. Braz Dent J.

[17] Fekrazad R, Nokhbatolfoghahaei H, Khoei F, Kalhori KA (2014). Pyogenic Granuloma: Surgical Treatment with Er:YAG Laser. J Lasers Med Sci.

[18] Chandna S, Kedige SD (2015). Evaluation of pain on use of electrosurgery and diode lasers in the management of gingival hyperpigmentation: A comparative study. J Indian Soc Periodontol.

[19] Magid KS, Strauss RA (2007). Laser use for esthetic soft tissue modification. Dent Clin North Am.

[20] Akbulut N, Kursun ES, Tumer MK, Kamburoglu K, Gulsen U (2013). Is the 810-nm diode laser the best choice in oral soft tissue therapy?. Eur J Dent.

[21] Kharadi UA, Onkar S, Birangane R, Chaudhari S, Kulkarni A, Chaudhari R (2015). Treatment of Oral Leukoplakia with Diode Laser: a Pilot Study on Indian Subjects. Asian Pac J Cancer Prev.

